# Concurrent Biocompatibility and Antimicrobial Functionality of Soft Diamond‐Like Carbon Coating for Healthcare Applications

**DOI:** 10.1002/adhm.202501224

**Published:** 2025-06-04

**Authors:** Abdul Wasy Zia, Alberto Tuñón‐Molina, Ioannis Anestopoulos, Miguel Martí, Iraklis‐Stavros Panagiotidis, Anam Ijaz, Ángel Serrano‐Aroca, Mihalis I. Panayiotidis, Martin Birkett

**Affiliations:** ^1^ Institute of Mechanical, Process, and Energy Engineering (IMPEE) School of Engineering and Physical Sciences Heriot‐Watt University Edinburgh EH14 4ST UK; ^2^ Biomaterials and Bioengineering Lab Department of Biotechnology Universidad Católica de Valencia San Vicente Mártir c/Guillem de Castro 94 Valencia 46001 Spain; ^3^ Translational Research Centre San Alberto Magno Universidad Católica de Valencia San Vicente Mártir c/Quevedo 2 Valencia 46001 Spain; ^4^ Department of Cancer Genetics, Therapeutics and Ultrastructural Pathology The Cyprus Institute of Neurology and Genetics Nicosia 1683 Cyprus; ^5^ Department of Comparative Biomedical Sciences School of Veterinary Medicine Mississippi State University Starkville MS 39762 USA; ^6^ Faculty of Engineering and Environment Northumbria University Newcastle upon Tyne NE1 8ST UK

**Keywords:** antimicrobial, biocompatibility, diamond‐like carbon, L929, MRSA, sputtering, surface coatings

## Abstract

This unprecedented study reveals the excellent antimicrobial performance of soft, hydrogen‐free, pristine diamond‐like carbon (DLC) coatings against life‐threatening methicillin‐resistant *Staphylococcus aureus* (MRSA). In addition, most previous studies have separately reported the biocompatibility and antimicrobial performance of DLC coatings, perhaps due to a nuance between antimicrobial performance and cytotoxicity. However, this work concurrently reports the biocompatibility of soft DLC with mouse fibroblast L929 cells and its antimicrobial performance against MRSA, where concurrent reporting is desirous for holistic material assessment, regulatory consideration, and enhanced safety and efficiency for biomedical applications. The soft DLC coatings are prepared with direct‐current, open‐field, magnetron sputtering, and characterized for structural, physical, mechanical, and electrical properties. The soft DLC coatings show excellent biocompatibility with 168 h leached extracts and antimicrobial performance after 24 h superior to copper, an established antimicrobial agent. This work proposes soft DLC as a safe material for concurrent biocompatibility and antimicrobial actions, as desired in most healthcare applications.

## Graphical abstract

Designing an engineering material that simultaneously promotes the growth of beneficial biological cells while eliminating harmful bacteria is often challenging. However, this study presents a unique carbon coating by manufacturing design capable of achieving both functions, demonstrating strong potential for various applications, including medical textiles, high‐touch surfaces, and orthopedic implants.

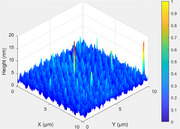

## 1 Introduction

Medical coatings are receiving wider attention for a range of healthcare applications, including medical: interventions, instruments, textiles, and structural surfaces. Considering an example of biomedical implants, these engineered surface coatings should be biocompatible to ensure medical safety and promote implant integration.^[^ ^1^ ^]^ In addition, the intervention surfaces should also be antimicrobial to mitigate bacterial transmission and prevent their growth and subsequent infections.^[^ ^2^ ^]^ Hospital‐acquired infections, including surgical site infections, significantly contribute to pathogen transmission, prevalence of new bacterial strains, and increased resistance of existing strains against drugs, where coagulase‐negative staphylococci, methicillin‐resistant *Staphylococcus aureus* (MRSA), and third‐generation cephalosporin‐resistant *Escherichia coli* are common examples.^[^ ^3^ ^]^ The World Health Organization has declared antimicrobial resistance a global threat to public health accounting for 1.27 million direct and 4.50 million indirect deaths globally in 2019,^[^ ^4^ ^]^ and 4.71 million associated and 1.14 million deaths were reported in 2021 directly due to bacterial resistance.^[^ ^5^ ^]^ The World Bank estimates that low‐impact and high‐impact antimicrobial resistance could globally result in £0.8 and £2.8 trillion loss in Gross Domestic Product by 2030 and an expected annual 3.8% shortfall by 2050.^[^ ^6^ ^]^

A range of polymeric,^[^ ^7^ ^]^ metal,^[^ ^8^ ^]^ ceramic,^[^ ^9^ ^]^ alloy,^[^ ^10^ ^]^ and carbon‐based^[^ ^11^, ^12^ ^]^ materials are used for biocompatibility and antimicrobial applications. A thin film coating of such engineering materials is deposited on medical interventions to promote their biocompatibility, antimicrobial performance, or other desired functioning. Diamond‐like Carbon (DLC) has been a highly popular choice due to its established biocompatibility,^[^ ^12^, ^13^ ^]^ higher hardness, Young's modulus, resistance against wear degradation, and ultimately lower friction coefficient. Under the umbrella of DLC, the coatings could be amorphous carbon, tetrahedral carbon, or graphite‐like structure, depending the sp^2^ and sp^3^ hybridization phase ratio,^[^ ^14^ ^]^ which regulates their physicochemical, mechanical, biological properties and tribological performance. DLC coatings are often doped with additional gaseous elements like nitrogen (N),^[^ ^15^ ^]^ hydrogen (H_2_), fluorine (F), etc, and a DLC nanocomposite^[^ ^16^ ^]^ are made with the addition of numerous types of nanoparticles, such as titanium (Ti),^[^ ^17^ ^]^ and silver (Ag),^[^ ^18^ ^]^ etc. to extend their biocompatibility. The recent scientific articles in 2025 report promoting DLC biocompatibility with the nanocomposition of silicon,^[^ ^19^ ^]^ Ag and Ti^[^ ^20^ ^]^ and doping with H_2_^[^ ^21^ ^]^ for artificial orthopaedic joints^[^ ^22^ ^]^ and other biomedical applications.^[^ ^23^ ^]^ Whereas, copper (Cu),^[^ ^24^ ^]^ zirconium oxide (ZnO_2_),^[^ ^25^ ^]^ etc. are demonstrated to extend the antimicrobial properties of DLC coatings. The doping or nanocomposition level significantly regulates the overall properties of DLC coatings including mechanical, tribological, biocompatibility, and antimicrobial performance. Metal nanocomposite DLC leads to suboptimal performance, where lower amounts of nanoparticles leads to ineffectiveness, while higher amounts pose a risk of cytotoxicity. Therefore, foreign elemental doping or nanocomposition into DLC coatings requires a balance between biocompatibility, antimicrobial performance, and toxicity. It is perceived that most studies have separately reported the biocompatibility and antimicrobial phenomenon of DLC coatings. The concurrent antibacterial action and biocompatibility of DLC are rarely reported, as DLC antimicrobial performance may increase with continuous addition of Bioact. Mater. such as Ag, however, excessively Ag nanocomospite DLC coatings become cytotoxic over a function of time.^[^ ^26^ ^]^ A recent study by Lu et al.^[^ ^27^ ^]^ suggests that the antimicrobial efficacy of DLC against *S. aureus and E. coli* increases with increasing Ag content in the coating matrix. However, the mouse osteoblast (MC3T3‐E1 Subclone 14) biocompatibility increased to an optimum value (≈7% in their studies) and then started to reduce with increased Ag contents. Hence, a proportional relationship between biocompatibility and antimicrobial performance becomes inverse beyond an optimum tertiary elemental doping or nanocomposition into a DLC matrix.

Besides cytotoxicity risks, the addition of metallic nanoparticles to DLC coatings also requires a compromise in mechanical hardness and wear rates, which significantly impact their biomechanical functioning, particularly for load‐carrying medical applications such as artificial hip and knee joints. The hydrogenated DLC coatings have lower^[^ ^28^ ^]^ hardness and higher^[^ ^29^ ^]^ wear rates when compared to hydrogen‐free DLC coatings. In addition, the compromise on hardness and wear rate continuously increases with the increase in hydrogen content in DLC,^[^ ^28^ ^]^ which impacts their mechanical performance significantly. Therefore, hydrogen‐free DLC have been desired for many engineering applications. Therefore, our group has recently been investigating the biomechanical performance of pristine DLC coatings (hydrogen‐free and without any elemental nanocomposition) produced with direct‐current open‐field magnetron sputtering and using argon as a processing gas, as a function of plasma deposition parameters to understand the correlation of carbon atomic bonds with mechanical properties and biocompatibility. We initially reported^[^ ^30^ ^]^ that relatively soft DLC coatings (relatively higher sp^2^ contents for a DLC coating but not a graphitic sp^2^‐enriched coating, a DLC coating having hardness yet more than steel) could be a better choice over hard DLC (relatively more sp^3^ contents in DLC coating) for biocompatibility of non‐load bearing applications. Following this, the recent study by Zhang et al.^[^ ^31^ ^]^ also validates that DLC with relatively more graphitic contents presents better compatibility against MG‐63 cells when tested for up to 5 days. The latest in vitro and in vivo studies on DLC toxicity investigated by Liao et al.^[^ ^32^ ^]^ suggest that excess graphitic contents also reduce DLC biocompatibility and promote an inflammatory response. Hence, it is perceived that there is an optimum amount of sp^2^ contents that can deliver superior biocompatibility.

L929 cells are widely and routinely used in cytotoxicity assays and assessment of biocompatibility of various materials in the sector of biomedical engineering, among others, since they are more sensitive to the toxic effect of different agents, when compared to human fibroblasts.^[^ ^33^ ^]^ In addition, L929 cells are considered as a conventional and reliable cell‐based system, as evidenced by various applications relevant to medical and biological research such as investigation of biocompatibility/cytotoxicity of dental implants^[^ ^34^ ^]^ and dermal fillers,^[^ ^35^ ^]^ as well as identifying and testing potential cancer therapeutic metabolic targets.^[^ ^36^ ^]^ MRSA is among the top bacterial threats as it typically transmits through contaminated surfaces. The epidemiological data from the Centers for Disease Control and Prevention in 2020 suggest that 15% of cases are from hospitals, 62% healthcare community‐onset, and 22% are community‐associated transmission. The MRSA biofilm causes recurring infections as bacteria prevail within biofilms and resist antibiotics by ≈1000 times more^[^ ^37^ ^]^ than planktonic counterparts.

This work concurrently investigates the biocompatibility of soft and pristine (hydrogen‐free) DLC coatings with murine fibroblast L929 cells and antimicrobial performance against MRSA. The hydrogen‐free DLC coatings used in this study are made with a sputtering method and characterized for atomic structure, contact angle, hardness, thickness, and conductivity. The biocompatibility and antimicrobial performance are then concurrently studied to assess the potential of soft DLC coatings in delivering combined biocompatibility and antimicrobial functions for a range of biological environments.

## 2 Results and Discussion

This section presents the structural, physical, electrical, mechanical, biocompatibility, and antimicrobial properties of hydrogen‐free soft DLC coatings.

### 2.1 Structural, Physical, Electrical, and Mechanical Properties of Soft DLC Coatings

**Figure 1:**
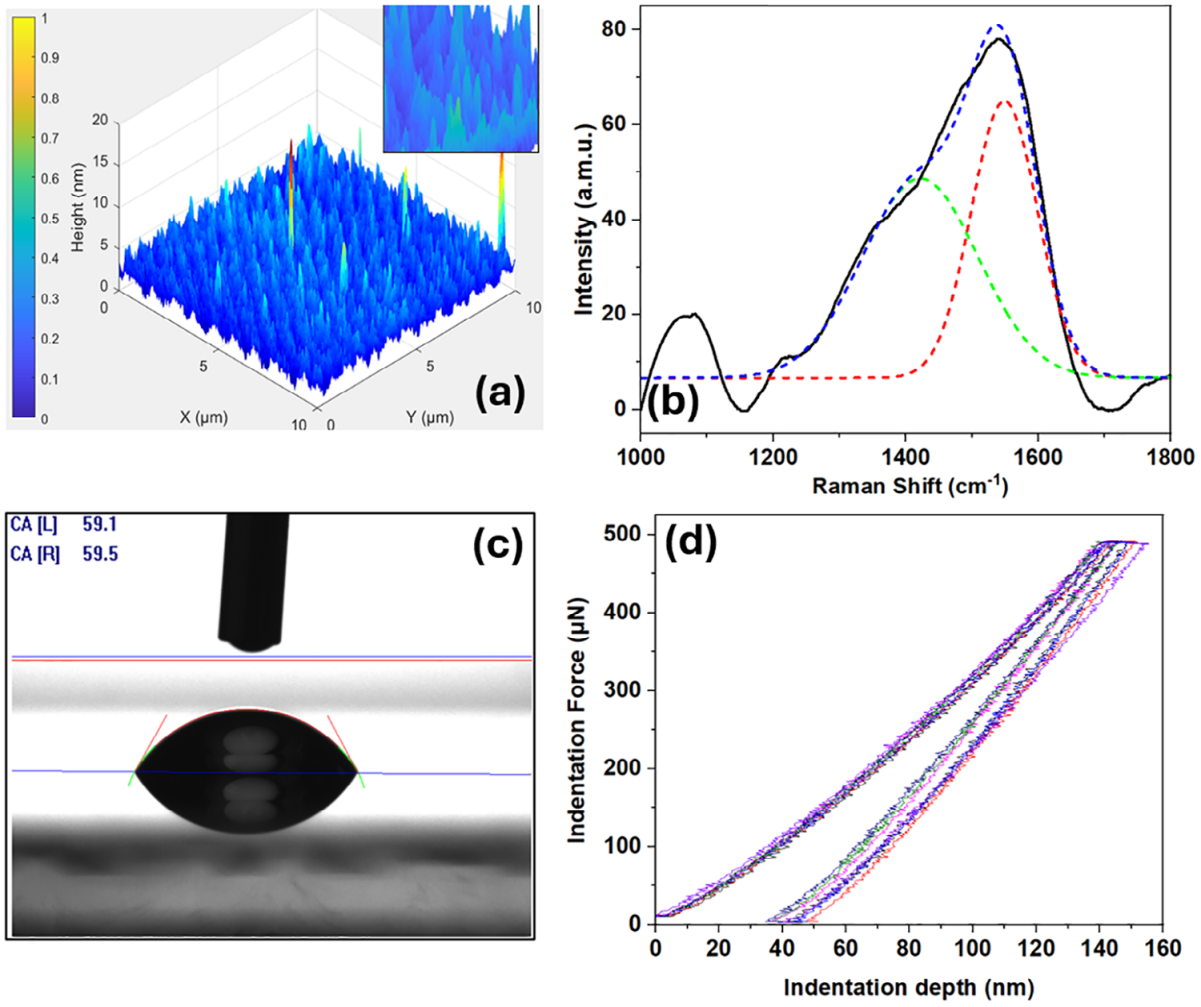
a) Surface roughness reconstruction of DLC coating (n = 3), the insert presents a closer view of surface asperities, b) a representative (n = 6) Raman spectrum of DLC coated glass substrate and its deconvolution, c) a representative image (n = 6) of DLC contact angle measurement with deionized water and d) nanoindenter load‐displacement curves (n = 9) for hardness measurement of DLC film.

The DLC coatings are recognised for lower surface roughness from a few nanometers to several nanometers. However, they have exhibited sharp surface asperities. The experimental atomic force microscopy for surface roughness measurement of DLC coating is shown in Figure S1 (Supporting Information). A Python‐based digital reconstruction of experimental images is presented in **Figure** **1**a. The DLC coating has an average roughness (R_a_) of 2.62 nm and root mean square roughness (R_q_) of 3.61 nm, as a few needles could be as high as 20 nm. A silicon wafer was used for laser calibration of the Raman spectroscope and the spectra is presented in Figure S2 (Supporting Information). The Raman peak was noted at 520 cm^−1^ which is consistent with literature reports.^[^ ^38^, ^39^ ^]^ The as‐received Raman spectra of DLC‐coated glass in given in Figure S3 (Supporting Information), while a processed spectrum, as observed in Figure 1b, presents a broad single peak which is a typical representation of amorphous carbon.^[^ ^40^ ^]^ Gaussian multipeak fitting suggests D peak position at 1421.8 ± 4.1 cm^−1^ and G peak position at 1548.9 ± 0.8 cm^−1^, and Full Width at Half Maximum of G Peak was 117.4. respectively. Referring to the three‐stage model,^[^ ^41^ ^]^ the G peak positions of 1520 cm^−1^ and 1575 cm^−1^ present carbon coating structure enriched with rings (a‐C) and chains (ta‐C), respectively. This suggests that the deposited coatings are amorphous and exhibit a highly disordered carbon atomic structure, a mixture of carbon rings and chains. The literature studies have reported G peak position for DLC coatings ranging from 1530 to 1560 cm^−1^ depending on deposition technology, deposition parameters, and characterization protocols.^[^ ^42^, ^43^ ^]^ The coatings are likely to have carbon sp^3^ bonds lower than 40%, referring to scientific literature.^[^ ^44^ ^]^ A small amorphous peak ≈1100 cm^−1^ appeared due to scattering from the glass substrate^[^ ^45^ ^]^ as the laser penetration was about twice the measured coating thickness of 235 ± 10 nm.

An average contact angle of 60 ± 2.5° was measured for the DLC coatings when tested with deionized water. Figure 1c is a representative example of contact angle measurement which suggests the hydrophilic nature of DLC coatings.^[^ ^46^ ^]^ Figure 1d presents load‐displacement curves from nanoindentation of the DLC coatings surface. The nano hardness was measured as 8.84 ± 1.2 GPa, reduced Young's modulus of 21.6 ± 3.9 GPa with a critical depth of 21.7 ± 2.7 nm. Steel variants normally have a hardness lower than 8 GPa, whereas, commercial grade DLC coatings usually have a hardness between 15 and 30 GPa,^[^ ^47^ ^]^ and research‐grade DLC coatings could be as hard as 80 GPa.^[^ ^48^ ^]^ Therefore, even though these DLC coatings are as hard as steel, they are “termed soft DLC” coatings due to their mechanical properties within the DLC family.

Referring to four‐probe electrical conductivity measurements, Cu has shown a conductive nature while bare and DLC‐coated glass specimens have shown electrical insulation. The Cu sheet resistance was 5.410 × 10^−02^ ± 3.301 × 10^−03^ Ω.□^−1^ and conductivity was recorded as 1.85E^+10^ ± 1.05E^+09^ S.m^−1^. Referring to the non‐conductive behavior of DLC, Yang et al.^[^ ^49^ ^]^ reported a significant increase in electrical insulation of steel with the application of DLC coating. In addition, the DLC resistivity varies from 10^4^ to 10^11^ Ω.cm depending on the sp^2^ and sp^3^ phase composition.^[^ ^50^ ^]^

### 2.2 Biocompatibility Studies of Soft DLC Coatings against L929 Mouse Fibroblasts

**Figure 2:**
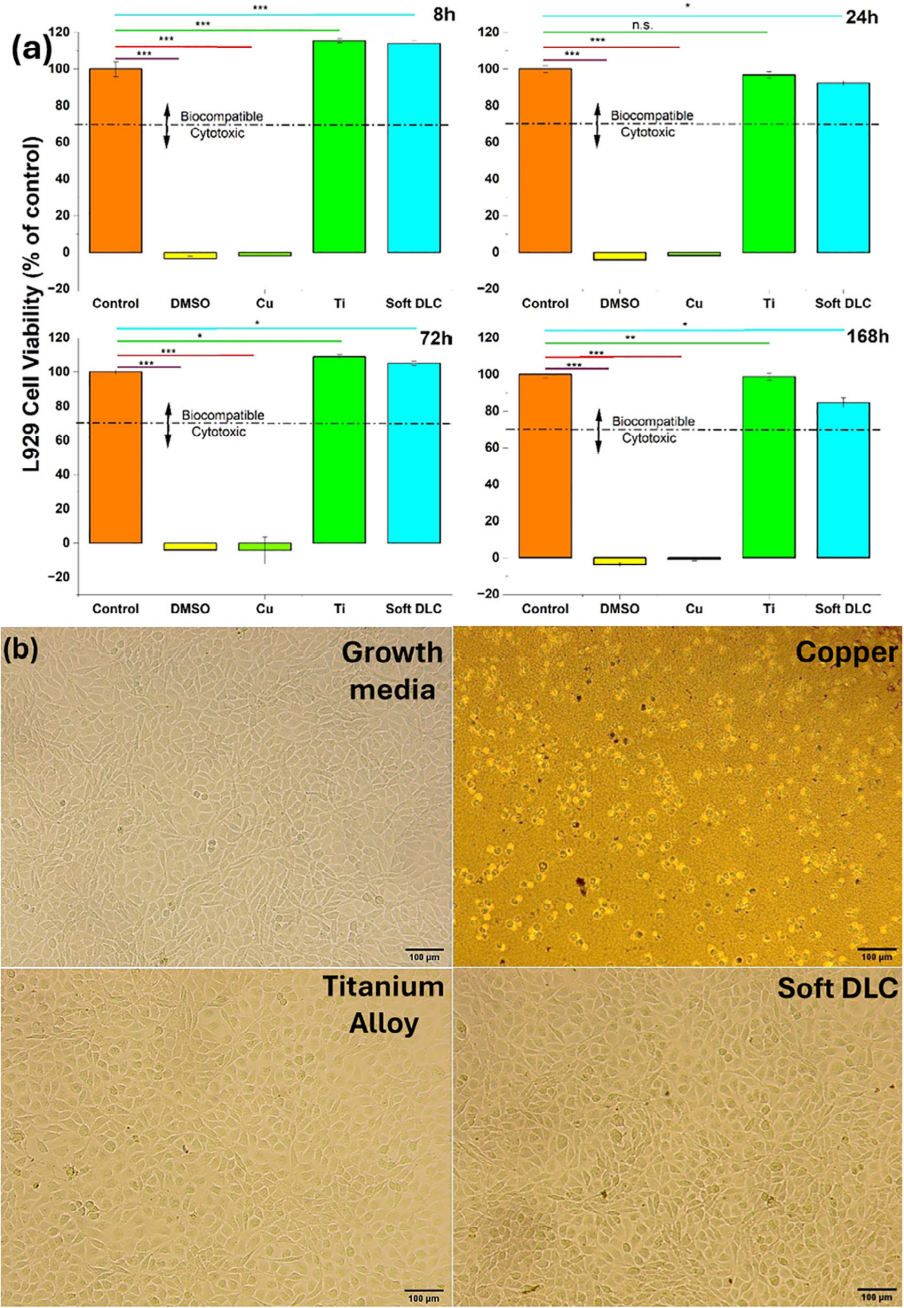
a) Viability levels of L929 mouse fibroblasts (means and standard deviations, n  =  3), following 72 h exposures with extracts obtained after 8, 24, 72, and 168 h ion leaching periods from soft DLC coatings, compared to i) negative (Growth media [Control], Ti) and ii) positive (10% of DMSO and Cu) controls. The statistical significance presented as *p < 0.05, **p < 0.01, ***p < 0.001, and ****p < 0.0001. b) Morphologies of L929 cells exposed to 168 h leached extracts for 72 h.

The viability levels of L929 cells following 72 h exposures with extracts obtained from DLC coatings after 8, 24, 72, and 168 h of ion leaching periods are presented in **Figure** **2**a. Viability as a function of time for negative (DMEM media) and positive controls (10% DMSO diluted DMEM), soft DLC and Ti alloy, and Cu as reference materials. An extreme cytotoxic effect was observed following treatments with 10% of DMSO and Cu leached extracts, as opposed to the effect of negative control samples, while soft DLC coatings exhibited high biocompatibility levels, comparable to those of Ti alloy‐derived extracts, even after incubations with extracts obtained from prolonged (168 h) leaching period. Specifically, although a 20% decrease in DLC biocompatibility rates was observed for 168 h leached extracts, a response probably due to increased carbon ions released in the culture media, viability levels were detected to be above 70%, indicative of a safe cytotoxic profile. Accordingly, the morphology of L929 cells was significantly altered upon exposure to Cu‐leached extracts, as evidenced by reduced confluency rates and cell shrinkage,^[^ ^51^ ^]^ both are associated with high cytotoxicity. The literature^[^ ^51^ ^]^ suggests L929 cells have linear and circular morphologies for living and dead cells, respectively. On the other hand, exposures to both Ti alloy and soft DLC‐derived ion leached extracts resulted in a safe cytotoxic profile, as morphology and confluency of L929 cells were not affected and were comparable to the effect of growth media (Figure 2b). Even though the soft DLC coatings are pure and free from any doping or composition of metal or ceramic elements, however, release of carbon ions is expected from the coating matrix. The literature suggests that the ion release rates are higher in crystalline and nanocomposite coatings than in amorphous materials, and the difference in release rates could reach 100% in 24 h. The higher ion release rates increase the probability of killing cells by rupturing their cell membranes. That could be a possible reason that the amorphous structure of DLC differentiates it from crystalline carbon allotropes^[^ ^52^ ^]^ for superior biocompatibility. The relationship between surface roughness and cell adhesion also contributes to biocompatibility. In general, the L929 cell size and DLC roughness have significant scale differences at the micro and nanometer. Therefore, the chances of contact killing mechanisms are reduced, except ion kinetics, where amorphous materials are well recognized for lower ion release rates than composites. In addition, the DLC has shown higher albumin/fibrinogen ratios^[^ ^53^ ^]^ which prevents thrombus formation, thus making DLC an attractive candidate for biomedical application when compared to other materials like carbon nitride, titanium nitride, titanium carbide, silicone elastomers, Poly(methacrylic acid), etc.

### 2.3 Antimicrobial Performance of Soft DLC Coatings Against MRSA

**Figure 3:**
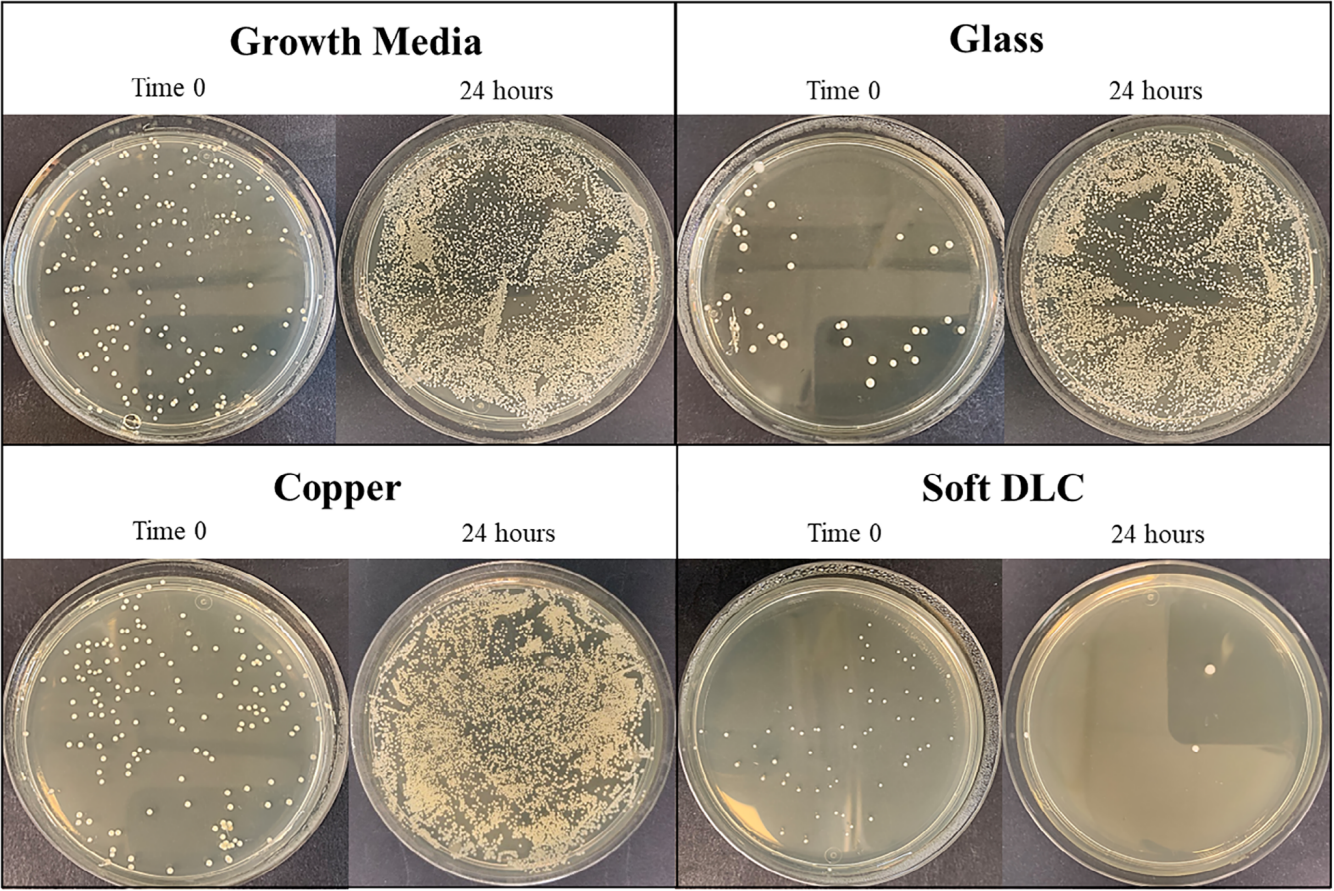
Representative (n = 3) images of plates with MRSA bacterial colonies from a single test search for each material at time 0 and after 24 h. Bacterial growth without being in contact with any material (growth media), glass, Cu bulk sheet, and soft DLC‐coated glass as the material under investigation. The dilution factor of each image is 10^−5^.

The detailed optical imaging of plates with MRSA bacterial colonies for each material at time 0 and after 0.5, 1, 3, 5, and 24 h are shown in Figure S4 (Supporting Information). **Figure** **3** presents the images of plates with MRSA bacterial colonies for each material at time 0 and after 24 h. Bacterial growth without being in contact with any material (growth media), glass as a negative control, Cu bulk sheet as a positive control, and soft DLC‐coated glass as the material under investigation. The increase in bacterial colonies after 24 h is visible for growth media, glass, and Cu. Whereas, a significant decline in MRSA bacterial colonies could be observed for the soft DLC coating.

**Figure 4:**
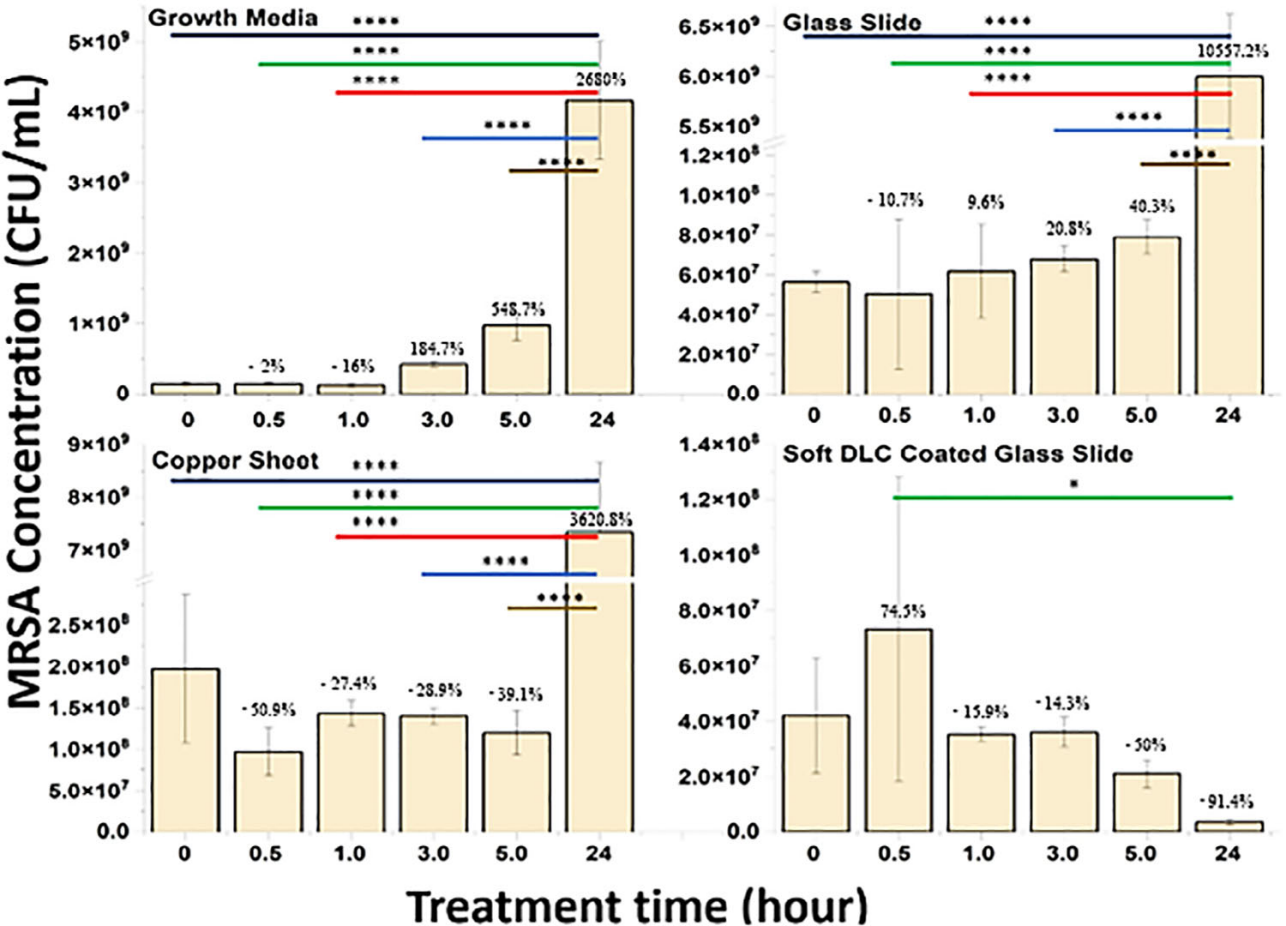
Methicillin‐Resistant *Staphylococcus aureus* growth or decay behaviors for bacterial growth media, glass, Cu, and soft DLC cultured up to 24 h (averages and standard deviations, n = 3). Statistically significant differences are indicated: ^*^ *p* < 0.05, and ^****^ *p* < 0.0001. Bacterial growth percentages are indicated above each column. Negative values represent a reduction in bacterial growth.

Whereas, **Figure** **4** presents the MRSA concentrations (CFU mL^−1^) in growth media (control), reference materials, and soft DLC solutions as a function of time up to 24 h. Statistically significant differences in MRSA are indicated with ^*^ *p* < 0.05, and ^****^ *p* < 0.0001 for all three types of materials and growth media control tested as a function of time (0 to 24 h). Referring to the antimicrobial performance from 0 to 24 h, It can be seen that the MRSA growth was statistically highly significant for growth media, glass and copper materials. Whereas, the MRSA grew in the early 0.5 h on soft DLC and then started to die off as a function of time. The soft DLC was the only sample that statistically demonstrated a significant reduction in MRSA after 24 h. Hence, the soft DLC has shown excellent antimicrobial performance, surpassing Cu, a widely used antimicrobial agent.

**Figure 5:**
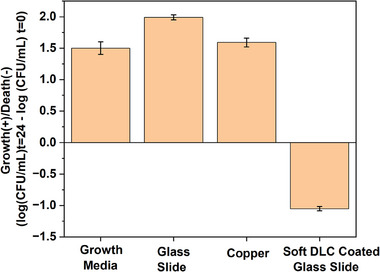
Antibacterial activity of growth media, glass, Cu, and soft DLC against the life‐threatening Methicillin‐Resistant *Staphylococcus aureus* after 24 h culturing in an orbital shaker (average of n = 3).

**Figure** **5** presents the MRSA growth or decay after 24 h at log scale. It can be seen that the growth media, glass and Cu all have at least +1.5 log (CFU mL^−1^) increase after 24 h. However, only the soft DLC coating has shown excellent antimicrobial performance of ≈1.0 log (CFU mL^−1^) MRSA reduction after 24 h. The soft DLC coating material clearly shows a potent antibacterial activity against MRSA decreasing bacterial concentration by more than one log. It is well‐known in microbiology that more than one logarithm reduction of bacterial concentration, which represents more than 90% of bacterial inactivation, is clearly accepted for a material to be considered antibacterial.^[^ ^54^ ^]^

The literature studies also suggest the excellent antimicrobial performance of hydrogenated and hard hydrogen‐free DLC coatings for healthcare applications. Prado et al.^[^ ^55^ ^]^ demonstrated that hydrogenated DLC‐coated ultra‐high molecular weight polyethene for hip joint applications was among the least adherent surfaces for nine different S*taphylococci* variants including five *S. aureus* clinical strains. Likewise, Kuwada et al.^[^ ^56^ ^]^ showed the reduced *S. aureus* attachment to a hydrogenated DLC coating deposited at the interior of a polyurethane tube. Whereas, the antimicrobial performance of hydrogen‐free hard DLC coatings against *S. aureus* is rarely found in the literature. Levon et al.^[^ ^57^ ^]^ demonstrated reduced adhesion of *S. aureus* to DLC micropatterns made with arc technology which is recognized for producing the hardest DLC coatings.^[^ ^58^ ^]^ The hydrophilic surfaces are usually more attractive for bacterial adhesion. However, DLC coatings exhibit nanoscale roughness as shown in Figure 2a. The same surface appears to have selective functions against L929 cells and MRSA colonies. As described above, the L929 cells are likely killed due to physical sliding, whereas the MRSA colonies during the shaking process and perform contact killing. In addition, the DLC coatings are recognized for low friction coefficients.^[^ ^59^ ^]^ Hence, it is anticipated that the lower surface roughness, reduced surface area per unit volume, and lower friction all contribute to bacterial inhabitation and biofilm formation. The decrease in *S. aureus* attachment to hydrogenated DLC was also observed in the studies of Kuwadai et al.,^[^ ^56^ ^]^ who suggests that sharp surface features may also have facilitated bacterial contact killing during the orbital shaking process. Likewise, Mi et al.^[^ ^60^ ^]^ also reported nano‐scale topologies as a bactericidal enabler by disrupting bacterial membranes. Beyond DLC coatings, a reduced surface roughness has also been reported as advantageous for antimicrobial actions in metals^[^ ^61^ ^]^ and bioactive ceramics.^[^ ^62^ ^]^ Hence, the significant decay in MRSA measured for soft DLC coatings in this study is likely due to the nanoscale roughness of soft carbon coating.

The hydrogen‐free soft DLC coating has shown surface roughness of 2.62 nm and hydrophilic behavior with a contact angle of 59°. The coating has shown a typical broad DLC spectrum with G peak position at 1548.9 ± 0.8 cm^−1^. The coating has a hardness of.84 ± 1.2 GPa, reduced Young's modulus of 21.6 ± 3.9 GPa, and has shown electrically insulating characteristics. Considering hydrogen‐free soft DLC biocompatibility with L929 cells and superior antimicrobial performance against MRSA, soft DLC could be a high‐potential material for a range of healthcare applications, particularly within contamination‐risk environments as they have shown 84% cell viability levels against control and about one log reduction in MRSA after 24 h of testing. The biocompatibility and antimicrobial results also suggest soft DLC as a potential coating material for contaminated structural surfaces, surgical tools and theatre equipment, textiles, and biomedical interventions like implants as they have shown a potential to resist bacterial growth superior to Cu material, for prolonged times.

## 3 Conclusion

This work unveils the concurrent biocompatibility and antimicrobial performance of hydrogen‐free soft DLC coatings. The soft DLC coatings are biocompatible for biomedical applications, but they should be antimicrobial as well to prevent infections. A soft hydrogen‐free DLC coating of ∼ 8 GPa hardness was made with direct‐current open field magnetron sputtering and tested for roughness, carbon hybridisation, contact angle, hardness and conductivity. The DLC coatings deposited in this work potentially hold electrical insulating characteristics, a contact angle of ∼ 60° and an IG peak position at 1548.9 ± 0.8 cm^−1^. The soft DLC coating showed high biocompatibility rates (above 85%), even after 72 h exposure of L929 fibroblast cells with 168 h derived leached extracts. In addition, the soft DLC coating has demonstrated superior antimicrobial performance against life‐threatening MRSA with ∼ 1 log (CFU mL^−1^) reduction after 24 h. Whereas the Cu control material appears to lose antimicrobial capacity after 5 h under the same testing conditions.

## 4 Experimental Section

### Specimens Preparation

Soft DLC coatings were deposited on Fisherbrand glass substrates (76 × 26 × 1 mm) for structural, physical, electrical, mechanical, biocompatibility, and antimicrobial studies. A silicon wafers (20 × 20 × 1 mm) were used for Raman spectroscopy, while bulk specimens of Ti alloy (Ti6Al4V/Grade 5/AMS4911/Brindley Metal, Powys, UK), Cu 101 (99.99% pure, Metal Supermarket, Gateshead, UK), and glass (all 26 × 19 × 1 mm, Fisherbrand UK) were used as controls for biocompatibility and antimicrobial studies. The hydrogen‐free soft DLC coatings were deposited using a Moorefield NanoPVD direct‐current, open‐field, magnetron sputtering system. Glass substrates were precleaned with propanol and dried with pressurized nitrogen before mounting on the substrate holder. The chamber was evacuated to 0.2 × 10^−3^ Pa. The coatings were deposited with a 99.9% pure graphite target of 50.8 mm diameter and 8 mm thickness, acquired from PI‐KEM, UK. The deposition parameters were target voltage and current of 557.2 V and 0.356 A, 1 rpm substrate rotation, 25 sccm argon flow rate, and working pressure of 0.839 Pa for a deposition time of 3 h to deposit a coating of 235 ± 10 nm. No substrate or target bias was applied for coating depositions. A Kapton tape was used for edge masking to produce a step for film thickness measurement. The coated samples were cut into a 26 × 19 × 1 mm size for biocompatibility and antimicrobial studies.

### Structural, Physical, Electrical, and Mechanical Studies

The coating thickness was measured with Bruker surface profilometer for scan length of 2 mm across the step produced by Kapton tape. The surface roughness was measured with Veeco Dimension 3100 atomic force microscopy. A surface area of 10×10 µm^2^ was assessed through 512 line scans in contact mode with a tip of 1 µm diameter. MATLAB software was used with Python programming for digital image processing to construct an enhanced visualisation of surface roughness. The point‐by‐point surface roughness data experimentally measured with AFM was imported into MATLAB in ASCII format. A Python code was made on the roughness governing equation, and the outcomes were validated with experimental data. Height points were used in a roughness governing equation to calculate the average roughness (R_a_) for cross‐validation with experimental data and reconstruction of a digital image with enhanced visualisation of surface asperities.

Raman spectroscopy was used to explore the carbon atomic arrangements in the DLC coatings. The experiments were conducted with a Horiba LabRAM HR using a HeNe gas laser at a wavelength of 632.8 nm, produced at 20 mW and the samples were focused with 50X lens. A silicon wafer was used for calibration and five measurements were performed for each carbon‐coated sample. The range of Raman shift measured was 200 to 800 cm^−1^ for silicon and processed for 1000 to 1800 cm^−1^ for DLC samples using OriginLab software. The as‐received spectrum was smoothed by 300 ponts of window using Savitzky–Golay filter. A baseline subtraction was performed with user selected 3‐points user selected 1000 cm^−1^, 1150 cm^−1^, and 1700 cm^−1^. The deconvolution analysis was performed with non‐leaning multipeak Gaussian fitting. The surface contact angle was measured with deionized water using a KRÜSS DSA 30 Droplet Shape Analyzer. A drop of 2 µL was used and 6 measurements were performed for each sample. The data was processed in Microsoft Excel for averages and standard deviations. The electrical sheet resistance (*R_S_*) of rectangular samples of 26 × 19 mm surface area was measured on a T2001A3‐EU four‐point probe system (Ossila Limited, Sheffield, UK). The electrical conductivity (*σ*) in S m^−1^ was determined according to Equation (1).^[^ ^63^ ^]^ Where *l* is the overall sample thickness measured with a digital calliper (Acha, Spain). The measurements were performed in triplicate to ensure reproducibility.

(1) $$\sigma =\frac{1}{R_{S}\;\times \;l}\;$$

Bruker's Hysitron Ti900 Nanoindentation platform was used for hardness measurements. The instrument works on a continuous stiffness measurement method and calculates the hardness as a function of force exerted per unit area. The hardness was measured in compliance with ASTM C1327–15 using a Berkovich tip and indentation was limited to less than 10% of coating thickness to exclude substrate effects. Three matrices of 3 × 3 indentations (a total of 27 indentations) were made, and data was processed in Microsoft Excel.

### Biocompatibility Studies

Biocompatibility/cytotoxicity tests of soft DLC coating extracts were performed against L929 mouse fibroblast cells. L929 cells were obtained from the German Collection of Microorganisms and Cell Cultures (GmbH), catalog number ACC 2, cultured in a humidified incubator at 37 °C and CO_2_ (5%) with DMEM high glucose media supplemented with streptomycin (100 µg mL^−1^), penicillin (100 U mL^−1^), 10% fetal bovine serum and streptomycin (100 µg mL^−1^). Extracts were prepared through the immersion of DLC coatings in 6‐well plates containing 6 mL of DMEM culture media for 8, 24, 72, and 168 h, while a slight agitation (5 sec) was performed during both the beginning and the end of incubations, in order to further promote ion leaching in the culture media. 6‐well culture plates were incubated for the above time points in a humidified incubator at 37 °C and CO_2_ (5%). At the end of each period of extract preparation, culture media of previously seeded L929 cells at a density of 2000 cells well^−1^ in 96‐well plated was removed and cells were exposed to the respective undiluted ion leached culture media for 72 h. In parallel, cells were also incubated for 72 h with DMEM culture media (Control) and Ti alloy derived ion leached extracts (negative controls), while DMSO (10%) and Cu ion leached extracts were used as positive controls. At the end of the 72 h incubations, the Alamar blues assay was performed as previously described,^[^ ^64^, ^65^ ^]^ while absorbance values were measured at 570 and 600 nm (reference wavelength). Finally, viability levels were expressed as % of untreated (Control) cells. An inverted Kern microscope, equipped with a digital camera and 10X lens was used for the acquisition of optical images.

### Antimicrobial Studies

The antibacterial activity of the soft DLC coatings against MRSA, COL,^[^ ^66^ ^]^ was evaluated by the colony‐forming units method.^[^ ^67^ ^]^ This MRSA strain was sourced from the Centro de Investigación y Tecnología Animal, Instituto Valenciano de Investigaciones Agrarias (CITA‐IVIA, Castellón, Spain). Bacterial growth media was used as a control, and glass and Cu specimens were used as reference materials. The subject materials were immersed in 25 mL of TSB (Scharlab) containing a dilution of ≈10^7^–10^8^ colony‐forming units (CFU mL^−1^) MRSA (OD_540_≈0.2). The dilution was cultured for 24 h at 37 °C and 240 rpm in an orbital shaker to mimic perfused hemodynamics. Three samples of 1 mL of bacterial dilution were collected before culturing in the orbital shaker (0 h) and at an instant of 0.5, 1, 3, 5, and 24 h to test the bacterial growth and possible antibacterial activity of subject materials. Serial dilutions for each replicate were prepared and 100 µL of each dilution was placed on a TSA (Scharlab) plate and spread with a cell spreader. Plates were incubated for 16 h at 37 °C. Colonies were counted to determine the concentration of viable MRSA cells, expressed as CFU mL^−1^, as a function of time from 0 to 24 h. Bacterial growth percentages were calculated using equation (2):

(2) $$\%\;Bacterial\;growth=\frac{\frac{CFU}{mL}\left(t\right)-\frac{CFU}{mL}\left(t=0\right)}{\frac{CFU}{mL}\left(t=0\right)}\;$$

where CFU mL^−1^ (t) represents the mean number of colony‐forming units per milliliter obtained from three biological replicates at each time point, and CFU mL^−1^ (t = 0) refers to the mean initial concentration of colony‐forming units per millilitre.

### Statistical Analysis

The data were presented as mean ± standard deviation (SD). The statistical analyses were performed using a one‐way analysis of variance (ANOVA), followed by Tukey's post‐hoc test for multiple group comparisons. The thresholds for statistical significance were set at ^*^ *p* < 0.05, ^**^ *p* < 0.01, ^***^ *p* < 0.001, and ^****^ *p* < 0.0001. The statistical analyses were performed using GraphPad Prism 10 (GraphPad Software, San Diego, CA, USA).

### Use of Artificial Intelligence

The authors confirm that they have not used any Generative AI tools to produce text or graphics in this work. Figure 2a was reconstructed from experimental data using standard MATLAB software with Python code, with full human instructions.

## Conflict of Interest

The authors declare no conflict of interest.

## Author Contributions

A.W.Z. performed conceptualization, investigation, funding acquisition, and writing − original draft. A.T.M. performed methodology, investigation, data curation, and formal analysis. I.A. performed investigation. M.M. performed methodology, investigation, data curation, validation, formal analysis, supervision, writing – review & editing. I.S.P. performed investigation. A.I. performed formal analysis, software, and visualization. M.I.P. performed supervision, resources, writing – review & editing. A.S.A. performed methodology, data curation, formal analysis, software, visualization, supervision, project administration, funding acquisition, validation, resources, writing – original draft, writing – review & editing. M.B. performed supervision, project administration, resources, funding acquisition, writing – review & editing.

## Supporting information



Supporting Information

## Data Availability

This work has produced new experimental data required to verify the DLC biocompatibility for L929 cells and antimicrobial performance against MRSA as a function of time. The data is available at https://doi.org/10.17861/b47fef2b‐84b0‐45b7‐9da5‐f19bd3f03e7d.
