# Supplementary material for: Concurrent Biocompatibility and Antimicrobial Functionality of Soft Diamond‐Like Carbon Coating for Healthcare Applications
Source: Adv Healthc Mater. 2025 Jun 4;14(19):2501224. doi: 10.1002/adhm.202501224 (PMC12304838; doi:10.1002/adhm.202501224)
Supplement: Supplementary file 1 — Supporting Information [file ADHM-14-0-s001.docx]

**Supporting information**

**Concurrent Biocompatibility and Antimicrobial Functionality
of Soft Diamond-like Carbon Coating for Healthcare Applications**

Abdul Wasy Zia^1*^, Alberto Tuñón-Molina^2,3^, Ioannis Anestopoulos^4^, Miguel Martí^2,3^, Iraklis-Stavros Panagiotidis^3^, Anam Ijaz^1^, Ángel Serrano-Aroca^2,3^, Mihalis I. Panayiotidis^4,5^, Martin Birkett^6^

^1^Institute of Mechanical, Process, and Energy Engineering (IMPEE), School of Engineering and Physical Sciences, Heriot-Watt University, Edinburgh, EH14 4ST, United Kingdom

^2^Biomaterials and Bioengineering Lab, Department of Biotechnology, Universidad Católica de Valencia San Vicente Mártir, c/Guillem de Castro 94, 46001 Valencia, Spain.

^3^Translational Research Centre San Alberto Magno, Universidad Católica de Valencia San Vicente Mártir, c/Quevedo 2, Valencia 46001, Spain

^4^Department of Cancer Genetics, Therapeutics and Ultrastructural Pathology,
The Cyprus Institute of Neurology and Genetics, Nicosia 1683, Cyprus.

^5^Department of Comparative Biomedical Sciences, School of Veterinary Medicine,

Mississippi State University, Starkville, MS 39762, USA.

^6^Faculty of Engineering and Environment, Northumbria University,
Newcastle upon Tyne, NE1 8ST, United Kingdom.

*Correspondence to: AW ZIA, Email: [a.zia@hw.ac.uk](mailto:a.zia@hw.ac.uk)


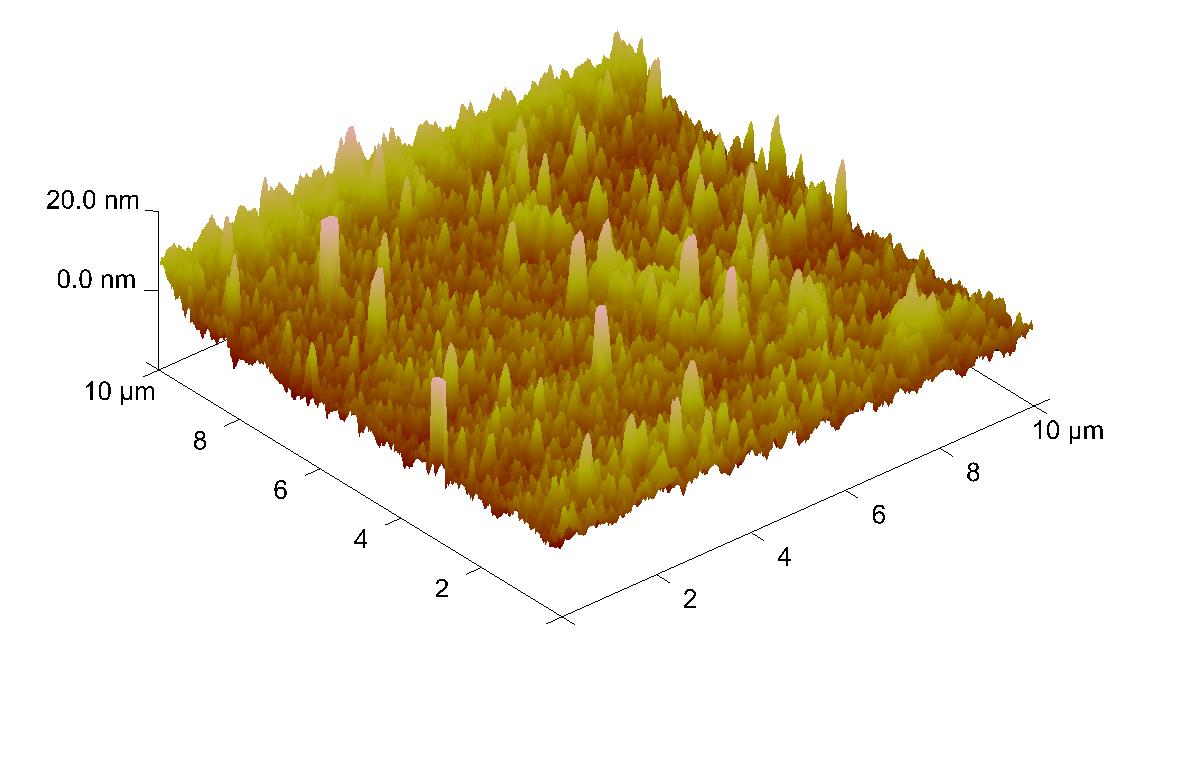


**Figure S2.** Atomic force microscopy of soft DLC coating for surface roughness measurement of 10×10 µm^2^


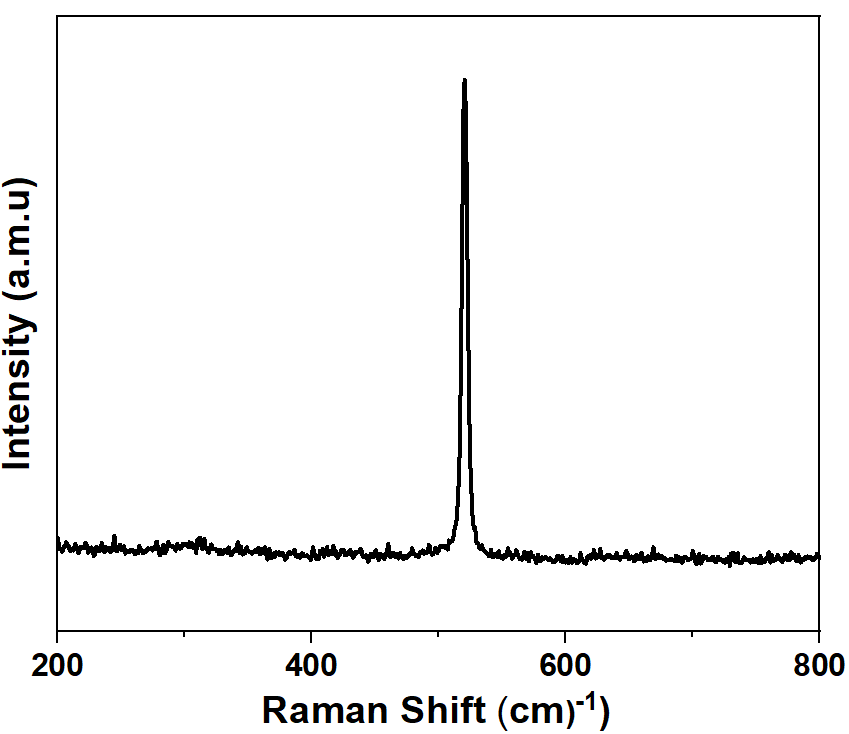


**Figure S2.** Calibration of Raman Spectroscopy on a silicon wafer.


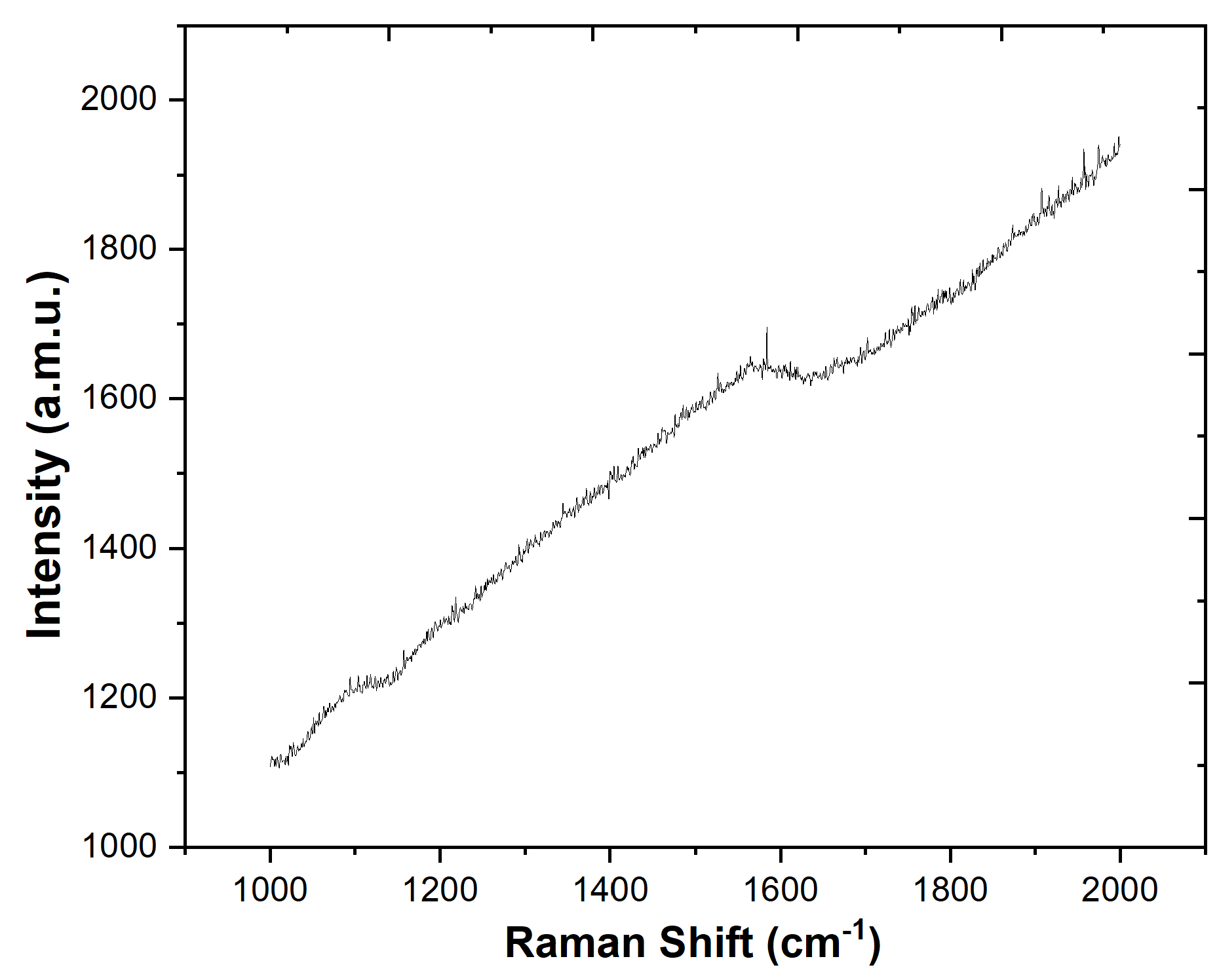


**Figure S3.** As received Raman Spectra of DLC coating


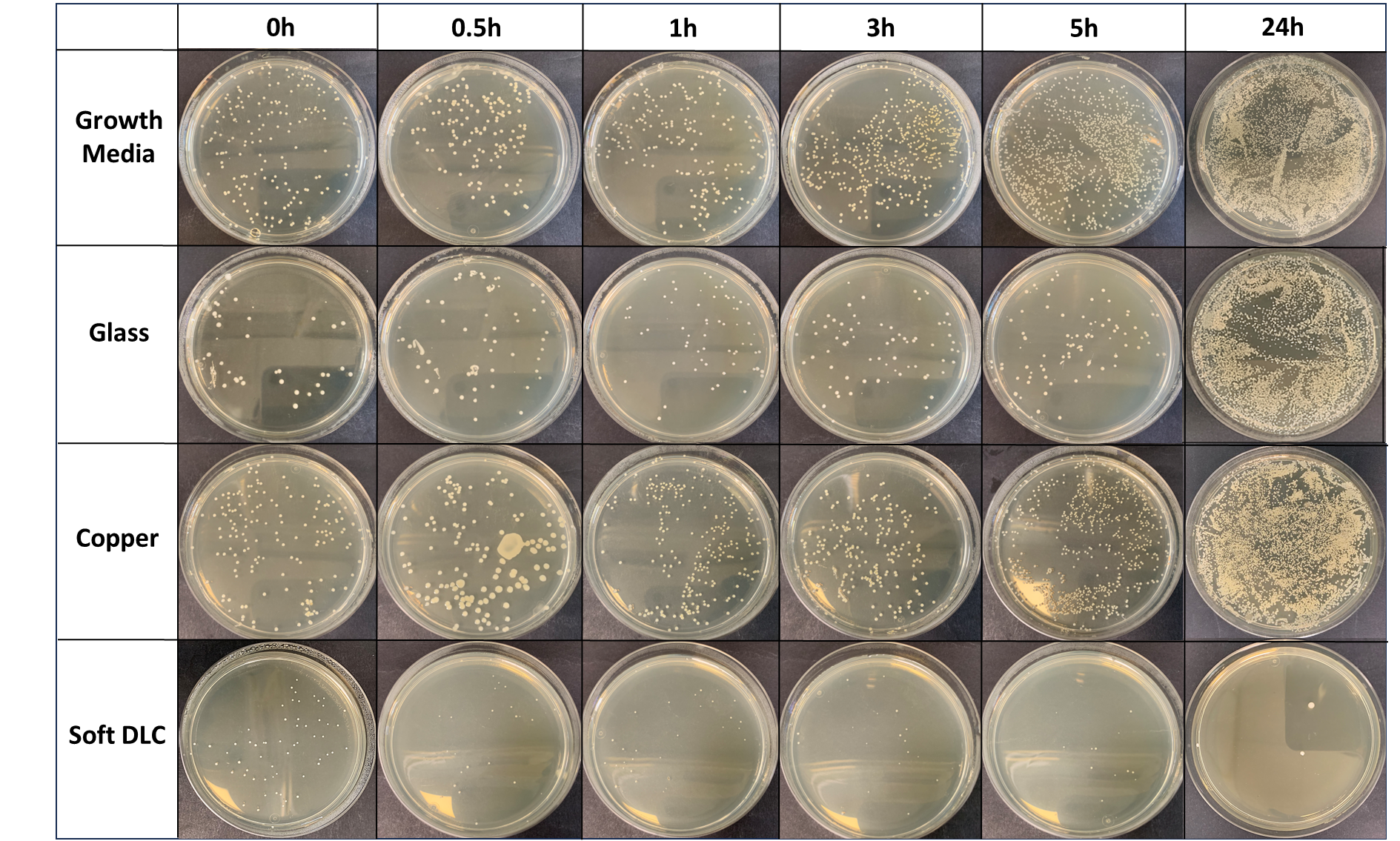


**Figure S4.** Images of plates with MRSA bacterial colonies for each material at time 0 and after 0.5, 1, 3, 5 and 24 h. Bacterial growth without being in contact with any material (growth media), glass, copper bulk sheet and soft DLC-coated glass as the material under investigation.The dilution factor of each image is 10^-5^.
